# Characteristics and mechanisms of resorption in lumbar disc herniation

**DOI:** 10.1186/s13075-022-02894-8

**Published:** 2022-08-23

**Authors:** Pengfei Yu, Feng Mao, Jingyun Chen, Xiaoying Ma, Yuxiang Dai, Guanhong Liu, Feng Dai, Jingtao Liu

**Affiliations:** 1 Department of Orthopaedic Surgery, Suzhou TCM Hospital Affiliated to Nanjing University of Chinese Medicine, Suzhou, 215009 People’s Republic of China; 2 Department of Orthopaedic Surgery, Kunshan Integrated TCM and Western Medicine Hospital, Suzhou, 215332 People’s Republic of China; 3 State Key Laboratory of Bioreactor Engineering & Shanghai Key Laboratory of New Drug Design, School of Pharmacy, East China University of Science and Technology, Shanghai, 200237 People’s Republic of China

**Keywords:** Lumbar disc herniation, Autoimmune response, Inflammation, Macrophages, Angiogenesis

## Abstract

Lumbar disc herniation (LDH) can be spontaneously absorbed without surgical treatment. However, the pathogenesis and physiological indications for predicting protrusion reabsorption are still unclear, which prevents clinicians from preferentially choosing conservative treatment options for LDH patients with reabsorption effects. The purpose of this review was to summarize previous reports on LDH reabsorption and to discuss the clinical and imaging features that favor natural absorption. We highlighted the biological mechanisms involved in the phenomenon of LDH reabsorption, including macrophage infiltration, inflammatory responses, matrix remodeling, and neovascularization. In addition, we summarized and discussed potential clinical treatments for promoting reabsorption. Current evidence suggests that macrophage regulation of inflammatory mediators, matrix metalloproteinases, and specific cytokines in intervertebral disc is essential for the spontaneous reabsorption of LDH.

## Introduction

Lumbar disc herniation (LDH) refers to the rupture of the fibrous annulus of the intervertebral disc, leading to the herniation of the nucleus pulposus and compressing the spinal nerve and cauda equina, causing an inflammatory response. The patient shows clinical symptoms such as pain and neurological dysfunction. With the change of work and life habits, the proportion of LDH patients has increased sharply and tends to be younger, which damages the physical and mental health of patients and becomes one of the main diseases that threaten human health [1]. Therefore, accurate diagnosis of the disease to obtain targeted treatment is particularly important. IDH diagnosis methods mainly include radiographic examination, myelography, computed tomography (CT), and magnetic resonance imaging (MRI). At present, MRI is the most effective method in the imaging diagnosis of disc herniation.

**Fig. 1:**
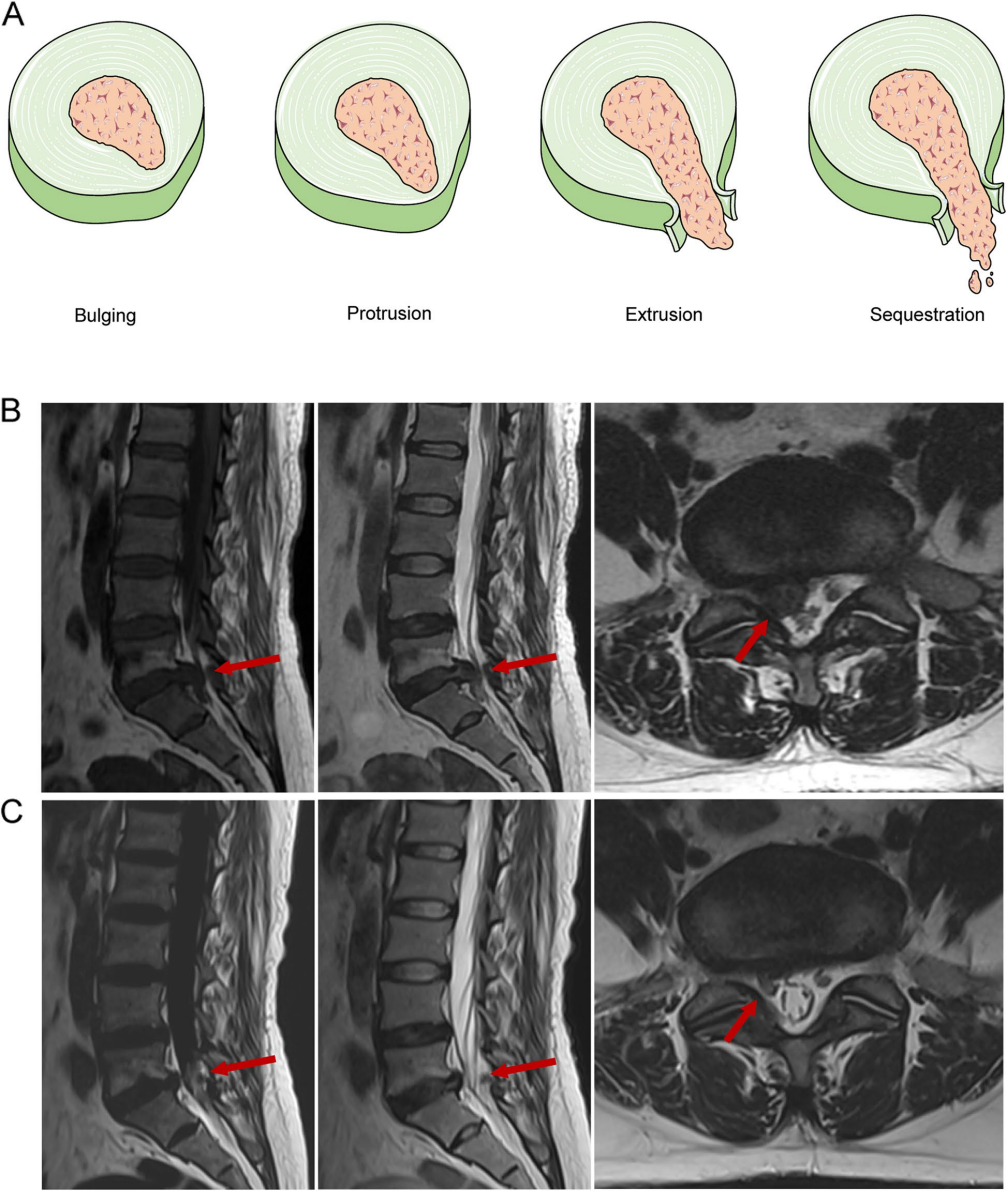
**A** The classification of LDH includes bulge, protrusion, extrusion, and sequestration. Elements in the image are taken from Servier Medical Art (https://smart.servier.com/). **B** Image from a 43-year-old female patient with LDH. The initial MRI showed that the L5/S1 intervertebral disc was hugely herniated, the center of the L5/S1 herniated intervertebral disc pushed the dural sac to the right, the right nerve root was compressed, and the dural sac was asymmetrically deformed. **C** After 1 year of conservative treatment, the L5/S1 disc herniation was significantly reduced, the protrusion was absorbed, and the dural sac was not significantly compressed or deformed

At present, the treatment strategies for LDH are divided into surgical treatment and conservative treatment. Conservative treatment is the first choice for most newly diagnosed LDH patients. The conventional course of treatment is at least 6 weeks, and the main forms include bed rest, drug therapy, exercise therapy, epidural injection, lumbar traction, and traditional Chinese medicine treatment [2–4]. Most of the LDH symptoms can be relieved by conservative treatment. In addition, through imaging examinations such as MRI and computerized tomography (CT), the herniated part of the intervertebral disc (IVD) in some patients shrank or even disappeared (Fig. 1B, C). Clinically, the phenomenon of spontaneous shrinkage or disappearance of a herniated lumbar IVD without surgical intervention is called reabsorption. At present, the spontaneous reabsorption of LDH has become a widely recognized clinical observation. What factors can predict reabsorption of herniated IVD? What conditions can induce or promote the reabsorption of herniated IVD? Related research work has been ongoing. In order to make more accurate decisions about conservative treatment for LDH, researchers urgently need to clarify the clinical features and biological mechanisms of the reabsorption of herniated IVD. Based on common clinical findings, this review focuses on the biological mechanism of IVD reabsorption, which provides a valuable basis for clinical prediction and diagnosis of IVD reabsorption, and for clinicians to rationally formulate treatment plans.

## Clinical manifestations of LDH reabsorption

Spontaneous absorption of LDH without surgical intervention was first reported in 1984 [5, 6]. This important discovery has provided a new way to study non-surgical therapy. In 1990, Saal et al. [7] selected 11 patients diagnosed with LDH by CT for conservative treatment in order to explore the natural history of LDH, and followed up with MRI. The results showed that 0–50% of the protrusions were absorbed in 2 patients, 50–75% in 4 patients, and 75–100% in 5 patients. In addition, the largest protrusions were the most resorbed, while the two smallest protrusions had the lowest reabsorption ratios. Saal believed that reabsorption usually occurs in the edema of nerve tissue, and the larger the protrusion, the more obvious the reabsorption. In 1996, Saal et al. [8] published a paper reviewing and analyzing the relevant clinical research reports on the reabsorption of LDH after non-surgical intervention, and discussed impacts of the types/locations of LDH, anatomical factors, histochemical factors, clinical characteristics and individual factors on the natural history of LDH. In recent years, researchers have paid more and more attention to the reabsorption of LDH. A meta-analysis showed that the average incidence of symptomatic LDH reabsorption was as high as 62–66% in 38 clinical studies reported in the past 30 years [9]. A recent retrospective analysis by our team showed that among 409 LDH patients, 320 patients received conservative treatment, and a total of 189 patients had protrusion reabsorption, accounting for 59.06% [10]. In addition, the North American Spine Society’s (NASS) Evidence-Based Clinical Guideline for the Diagnosis and Treatment of Lumbar Disc Herniation with Radiculopathy [11] pointed out that with the advancement of the natural history, most patients’ herniated IVD can spontaneously shrink or degenerate. Numerous studies have shown that as the degree of herniation decreases, clinical symptoms also improve. In conclusion, the phenomenon of LDH reabsorption is not accidental, and the clinical symptoms of most patients can be relieved or even disappeared under the condition of non-surgical treatment or no treatment.

## Clinical and imaging features of LDH prone to reabsorption

IVD reabsorption is common in LDH patients. At present, the treatment of LDH can be divided into conservative treatment and surgical treatment. Which treatment is used depends on the imaging features of the herniated IVD and other clinical factors. The clinical treatment decision for LDH needs to take into account the type of disc herniation, the size of the protrusion, the composition of the herniated IVD, and the enhancement of the MRI signal around the protrusion.

### The types of LDH

MRI imaging divides the morphology of LDH into bulging, protrusion, extrusion, and sequestration [12] (Fig. 1A). Cases of natural absorption of these four types of LDH have been reported. It is generally accepted that extrusion and sequestration of LDH are more prone to regression than other types. In 2014, Chiu et al. systematically evaluated the probability of reabsorption of different types of LDH. Most LDH can be absorbed spontaneously after conservative treatment. Extrusion and sequestration LDH had higher regression rates than bulging and protrusion LDH, because both IVDs tend to penetrate the annulus fibrosus and the posterior longitudinal ligament, thereby being exposed to the systemic circulation in the epidural space [9, 13]. Extrusion and sequestration are the most damaging types of LDH. Due to the severe mechanical compression and inflammatory stimulation of the dural sac and nerve root, the extrusion and sequestration types often have typical radicular symptoms, and even spinal cord compression. Therefore, for the extrusion and sequestration of LDH, we recommend conservative treatment as the first choice in terms of risk factors and safety. In conclusion, the urgent questions we need to answer are as follows: (1) Is there unacceptable neurological damage or risk of cauda equina syndrome during the reabsorption process? (2) Is the improvement of clinical symptoms after conservative treatment temporary or persistent? (3) Will the symptoms relieved by conservative treatment recur? According to literature reports [10], we can conclude that if the early clinical symptoms (e.g., pain and numbness of lower limbs) of patients with squeezing and sequestering LDH can be effectively improved, conservative treatment is relatively safe. In order to avoid the risk of irreversible nerve root damage and cauda equina syndrome, we must observe the patient frequently during conservative treatment to minimize the risk. However, if the following conditions occur, surgical intervention should be performed immediately: (1) the symptoms are not significantly relieved after 3–6 months of conservative treatment; (2) the symptoms are aggravated by conservative treatment; (3) the clinical manifestations of cauda equina syndrome. Our clinical study also found that [10], if patients were closely followed and observed with reference to the above conditions, only 21.76% of patients with extruded and sequestrated LDH eventually received surgery. In addition, from the follow-up observation of more than half a year, no irreversible nerve root injury and cauda equina syndrome occurred in the conservatively treated patients, which means that clinicians should fully consider the possibility of LDH regression when making a diagnosis/decision and conservative treatment should be given priority in the absence of surgical indications.

### The size of LDH

In clinical diagnosis, the clinician will decide whether or not surgical intervention is required based on the size of the patient’s herniated disc. Generally speaking, the probability of surgery is greater when the herniated disc is larger. However, recently Gupta et al. pointed out that it is not feasible to use the size of the intervertebral disc herniation (as a percentage of the spinal canal area) to predict whether surgery is required after 6 weeks of conservative treatment [14, 15]. Similarly, cases of reabsorption of large herniated discs have been reported many times [14, 16, 17]. Therefore, in addition to patients with cauda equina syndrome and motor nerve function defects that require emergency surgery, regardless of the size of the disc herniation, doctors need to consider conservative treatment when making treatment decisions, which can largely avoid the economic burden and side effects of surgery.

### The composition of herniated disc-cartilage endplate

#### The components of the herniated IVD nucleus pulposus, annulus fibrosus, and cartilage endplate

The composition of the IVD may affect reabsorption. The herniated disc tissue contains annulus fibrosus, cartilage endplate, and nucleus pulposus [18]. Resorption may be favored when the herniated disc contains most of the nucleus pulposus. Iwabuchi et al. [19] used plain MRI to predict the effect of herniated disc composition on resorption. According to the signal intensity of T1 weighting and T2 weighting, the herniated disc components are divided into 5 types. The components of type 1–5 herniated IVD are nucleus pulposus, annulus fibrosus, mucinous tissue, hyperplasia of granulation tissue, and part of annulus fibrosus. The research group speculated that type 1 and type 5 intervertebral disc herniation tends to be reabsorbed by analyzing cases, especially type 1, whose tissue composition is mainly the nucleus pulposus, with high water content and low degree of degeneration. Therefore, it is more conducive to the ingrowth of blood vessels and the dehydration of tissue components, thus promoting the reabsorption of the herniated IVD. In addition, most of the sequestered LDH, which is widely considered to be prone to reabsorption, is dominated by the nucleus pulposus.

Another study suggested that cartilage tissue in protrusions may inhibit reabsorption. Schmid et al. showed that changes in the bone marrow signal intensity of the vertebral endplates suggested the presence of cartilage tissue in extruded LDH [20]. Part of the tissue in the protrusion containing cartilage fragments showed Modic changes, that is, the lumbar endplates and sublumbar bone showed abnormal signal changes via MRI [20]. Modic changes are divided into 3 types. Modic changes in LDH patients are mostly type II [21]. Concomitant Modic changes are closely related to LDH containing hyaline cartilage. Less capillary formation and macrophage infiltration are present in hyaline cartilage with Modic alterations. The failure of spontaneous absorption of LDH is mainly related to cartilage protrusion [22]. The results of an in vivo trial demonstrated abundant neovascularization and inflammatory cell infiltration in corneas implanted with annulus fibrosus compared with those implanted with endplates, whereas corneas with cartilaginous endplates showed inhibition of angiogenesis [23]. Lama et al. found that the tissue of hyaline cartilage fragments had few phenomena such as swelling, proteoglycan reduction, and inflammatory cell invasion in saline. In contrast, the nucleus pulposus and annulus fibrosus tissue rapidly swell in saline, resulting in increased pore size, which in turn promoted the massive loss of proteoglycans that inhibit vascular growth [24]. More preclinical and clinical studies are needed to clarify the relationship between cartilage endplates and LDH reabsorption. The effect of the protrusion component on reabsorption may depend primarily on whether it favors vascularization, whereas hyaline cartilage fragments tend to resist revascularization and resorption. In conclusion, the above findings suggest that if MRI shows the protrusion is predominantly nucleus pulposus, it may favor resorption; however, a high proportion of cartilaginous endplates may make LDH difficult to relieve, and thus conservative management may not be effective.

#### MRI signal enhancement around herniated disc

The dissociated intervertebral disc to the epidural space can cause an autoimmune reaction, which leads to an inflammatory reaction, and the formation of surrounding granulation tissue, which is manifested as ring enhancement, while the center-free intervertebral disc has no enhancement, which is called “bull’s eye sign.” The herniated intervertebral disc tissue in the membrane was examined by MRI, showing macrophage infiltration and angiogenesis in the herniated tissue; Gd (gadolinium diethylene triamine pentaacetic acid)-enhanced MRI image showed that the signal ring around the protrusion was enhanced. And the greater the thickness of the edge enhancement, the higher the signal enhancement. The obvious edge enhancement of the herniated tissue suggests the possibility of spontaneous absorption, which is also considered to be an important factor in evaluating the spontaneous regression of herniated discs [25–27]. Ring enhancement (ring), or bull’s eye sign, is believed to be related to the vascularization of lumbar disc herniation and the formation of inflammatory granulation tissue [28, 29], and the neovascularization and inflammatory response of the herniation are the key factors for reabsorption [30]. Therefore, the enhancement around the protrusion can be used as the imaging manifestation of the formation of new blood vessels and inflammation in the prominent tissue, which can predict reabsorption. However, due to current clinicians’ understanding of the phenomenon of reabsorption, the economic conditions of patients, and the adverse effects of injection of contrast enhancers, it is still difficult to use enhanced MRI to diagnose lumbar disc herniation.

Although there is clinical evidence of spontaneous LDH absorption, the difference in efficacy between conservative and surgical treatment is still controversial. Compared with other LDH types, patients with squeezing and sequestered LDH are preferentially given conservative treatment measures. Because these two types of LDH reabsorb more easily and faster than the other types. However, if the dural sac and nerve root are compressed due to the large protrusion, and the radicular pain of the lower extremity is aggravated, non-surgical treatment such as medicine and physiotherapy may be difficult to relieve the symptoms in the acute phase, and the larger herniated intervertebral disc is prone to recurrence. In such cases, clinicians may prioritize surgical intervention. Several large cohort studies, such as the Maine Lumbar Spine Study [31], the Spine Patient Outcomes Research Trial (SPORT) [32], and the Hague Spine Intervention Outcomes Study Group [33], have shown that early surgical intervention in LDH relieves clinical symptoms faster than conservative treatment. However, in the long term, the results of surgical treatment were almost identical to those of conservative treatment. In addition, predicting the occurrence of reabsorption needs to be combined with MRI imaging, and the presence of Modic changes in some tissues may mean a lower probability of reabsorption. A bull’s eye sign with annular enhancement around a herniated IVD may be an important indicator of reabsorption. However, the incidence and mechanism of reabsorption have not been clearly elucidated, and it is still difficult for clinicians to predict the likelihood of spontaneous regression of LDH and which patients are more likely to benefit from conservative treatment. Therefore, more large-scale and randomized experiments are needed in the future to better determine the criteria for clinical decision-making, for example, we can explore more intuitive and uniform MRI imaging features or reliable biomarkers.

## The mechanism of LDH reabsorption

At present, we mainly use imaging features to preliminarily judge the possibility of reabsorption, and the biological mechanism of reabsorption has not yet been elucidated. Three possible mechanistic hypotheses have been reported in the literature, which may be jointly involved in the resolution and disappearance of LDH. The first mechanism is retraction of the protrusion, which may occur without separation of the protrusion from the annulus fibrosus [34]. The second mechanism is the gradual dehydration and contraction of the herniated nucleus pulposus, which in turn causes the protrusions to retract into the annulus fibrosus [35]. A third mechanism, which has received extensive attention, states that fragments of herniated IVD enter the epidural space, triggering an autoimmune response including inflammatory cell infiltration and neovascularization. The autoimmune system recognizes LDH protrusions as “foreigners” in the vertebral epidural vascular space, which in turn triggers a cascade of inflammatory responses including neovascularization, matrix protease activation, increased levels of inflammatory mediators, phagocytosis of inflammatory cells, and enzymatic degradation [5, 13, 36]. This is supported by multiple clinical data, histopathological studies, and animal experiments [37–40]. This article will focus on the third mechanism and discuss the physiological changes such as macrophage infiltration, inflammatory cytokine accumulation, enzymatic degradation response, and neovascularization caused by autoimmune responses during LDH reabsorption.

### Immune privilege

Immune privilege is the condition in which selected immune responses are suppressed or excluded in certain organs [41]. Normal disc tissue is mainly composed of annulus fibrosus (AF), cartilage endplate (CEP), and nucleus pulposus (NP). AF is a ligamentous laminar structure wrapped around NPs, mainly composed of type I collagen fibers. CEP consists of a small amount of hyaline cartilage located between the vertebral endplates and the NP. NP is a hydrated structure, mainly composed of proteoglycans interspersed in an irregular network of type II collagen fibers. In the early stages of human life, NP consists of large numbers of vacuolar notochord cells and small chondrocyte-like cells. However, as IVD matures, the notochordal cells in the NP disappear, and the NP transforms from a notochordal structure to a tissue embedded with small chondrocyte-like cells [42–44]. Normal IVD is avascular, with a unique structure that isolates the nucleus pulposus from the host immune system. Normal and stable IVD inhibits immune cell and cytokine infiltration. The immune privilege of IVD is attributed to the blood-NP barrier as well as the local expression of Fas ligand (FasL). The lymphocytes accumulation in the regional nodes after exposure of the autologous NP [45], IVD lymphocyte accumulation in the annulus fibrosus injury [46], and deposition of human LDH immunoglobulin and complement membrane attack complexes all support this theory [47–49]. FasL-Fas maintains IVD immune privilege and prevents human IVD angiogenesis by inducing apoptosis of immune cells and vascular endothelial cells through a complex signaling pathway [50, 51]. In addition, when the blood-NP barrier is compromised, such as when a herniated IVD is exposed to the immune microenvironment, an autoimmune response is triggered, leading to multiple pathological processes such as neovascularization and immune cell infiltration. Inflammatory cytokines (e.g., tumor necrosis factor-α (TNF-α) and interleukin-1β (IL-1β)) secreted by immune cells regulate matrix metalloproteinases (MMPs), disintegrins, and a disintegrin and metalloproteinase with thrombospondin motifs (ADAMTS), which accelerate the breakdown of the extracellular matrix (ECM) and enhance the recruitment of immune cells to this area, thereby maintaining and promoting inflammation. In addition, CD4+ T cells are present within IVD. CD4+ T cells can be divided into Th1 and Th2 subtypes according to the cytokines secreted. Th2 cells mediate humoral immune responses by producing IL-4, IL-5, IL-6, IL-10, and IL-13. Th1 cells enhance cellular immunity by producing interferon-γ (IFN-γ) and IL-2. One study showed that IVD preferentially expresses Th2 cytokines, which may be another factor contributing to IVD immune privilege [52]. Exposure of lumbar IVD to the epidural space may increase IL-12 concentrations, thereby altering the expression patterns of Th1 and Th2 cytokines [52].

Traditionally, the imbalance of immune privilege caused by intervertebral disc lesions is a harmful immune response, which is considered to be an important factor leading to intervertebral disc degeneration and sciatica. Although infiltration of inflammatory cells/growth factors/cytokines and immune cascades may contribute to disc degeneration, certain specific immune cells may facilitate regeneration of injured disc tissue and restoration of immune privilege. The functions of such immune cells may be to eliminate cellular debris and secrete anti-inflammatory cytokines [53, 54]. To be sure, IVD immune privilege supports the inflammatory response during spontaneous LDH reabsorption. When NP tissue is squeezed out of the epidural space, it will cause an autoimmune response, leading to immune cell infiltration, and the recruited immune cells will interact with the intervertebral disc cells to secrete various factors to promote the resorption of the intervertebral disc.

### Macrophage is a key immune regulator that triggers LDH reabsorption

Macrophage infiltration and activation are critical steps in the process of reabsorption. Infiltration of macrophages in human LDH has been extensively demonstrated. Djuric et al. found abundant infiltrating macrophages in extruded and sequestered LDH. The number of macrophages in extruding LDH is higher than that in bulging LDH, which may be due to the fact that it has a larger surface for macrophages to adhere to, which in turn promotes the protrusions to be more absorbed [55, 56]. In herniated disc fragments, there are greater expression of IL-12 and IFN-γ compared to bulging discs that remain contained within the disc space by an intact annulus fibrosus [57]. IFN-γ produced by Th1 lymphocytes recruits and activates more macrophages [52]. Specifically, contact of NP tissue in LDH with the systemic circulation leads to lymphocyte activation and secretion of IFN-γ, which in turn promotes macrophage recruitment. Elevated IFN-γ expression in herniated discs may represent a specific immune response against herniated NP tissue [58]. These findings suggested that the mode of immune activation in LDH involves macrophage infiltration and activation. To more clearly illustrate the relationship between LDH reabsorption and macrophages, we will sequentially introduce the mechanisms driving the recruitment of macrophages in herniated IVD, the pro-/anti-inflammatory effects of macrophages in herniated IVD, and the crosstalk between macrophage-induced inflammatory responses and matrix metalloproteinase activation/neovascularization.

#### Mechanisms that drive recruitment of macrophages in herniated IVD

The expression of some cytokines and chemokines in IVD is an important factor in macrophage recruitment. Chemokines, such as monocyte chemoattractant protein-1 (MCP-1), monocyte chemoattractant protein-3 (MCP-3), monocyte chemoattractant protein-4 (MCP-4), chemokine (C-C motif) ligand 5 (RANTES), macrophage Inflammatory Protein-1 α (MIP-1α), and interferon-γ–inducible protein10 (IP-10), have been extensively validated for expression in human IVD [37, 59]. Human IVD can spontaneously produce inflammatory mediators that help recruit immune cells in a paracrine manner, particularly MCP-1, CC chemokine, and IL-8, which mediate macrophage chemotaxis and pro-angiogenesis [59, 60]. A rabbit model of LDH suggested that intervertebral disc cells may produce TNF-α, IL-1β, and MCP-1 immediately after LDH, which promotes macrophage infiltration and LDH reabsorption [61]. When macrophages are recruited to the protrusions, the cytokines they secrete further promote their recruitment in an autocrine manner. Herniated IVD contains high levels of TNF-α, IL-1β, IL-6, IL-8, prostaglandin E2 (PGE2), and nitric oxide (NO) [58, 62]. Most of these cytokines are the products of macrophages, which can promote the activation and differentiation of lymphocytes and can also recruit more macrophages, activate phagocytosis, and secrete proteolytic enzymes. Haro et al. detected the chemokines MCP-1 and MIP-1α in surgically resected herniated nucleus pulposus samples, which are highly expressed in macrophages, fibroblasts, and endothelial cells, and possibly activate/recruit macrophages in a paracrine or autocrine manner [37]. To further elucidate the effect of chemokines on intervertebral disc reabsorption, the research team then established a rat autograft model with the introduction of lumbar dura mater. Their results showed that human recombinant MCP-1 could accelerate the regression process of herniated nucleus pulposus [63].

Thus, these studies suggested that chemokines, especially MCP-1, are important mediators of macrophage infiltration into IVD (Table 1). Biomolecules that drive chemokine production by IVD and macrophages include thymic stromal lymphopoietin (TSLP), inflammatory factors (e.g., TNF-α, IL-1β), and MMP3. In normal IVD, endogenous TGF-β restricts TSLP expression by inhibiting NF-κB activation. TGF-β1, -β2, and -β3 pan-neutralizing antibody induced TSLP expression in mouse intervertebral disc tissue. NF-κB is an important transcription factor required for TSLP expression. TGF-β signals through the Smad protein family, which interacts with many intracellular signaling pathways, including the NF-κB pathway, and induces activation or repression of gene expression in a cellular microenvironment-dependent manner [71]. TGF-β (e.g., TGF-β1), present in bone and cartilage, binds to extracellular matrix molecules (ECM) in complexes and is activated by mechanical traction to signal healthy intervertebral disc cells. The herniated disc tissue may lose the opportunity to contact the extracellular matrix, so the NF-κB in the tissue cannot respond to the signal released by the activation of the TGF-β complex to upregulate TSLP levels [67, 72–74]. Upregulated TSLP induces the expression of MCP-1 in mouse intervertebral disc cells through the PI3K/Akt signaling pathway [64, 67]. Three isoforms of TGF-β (TGF-β1, TGF-β2, TGF-β3) are distributed in the intervertebral disc tissue [72]. Of the three isoforms, TGF-β1-mediated signaling is the most studied in the intervertebral disc. According to the present research reports, we speculate that TGF-β1 is involved in inhibiting the expression of TSLP in herniated intervertebral discs [75, 76], but the molecular mechanism of TGF-β1-mediated TSLP expression still needs to be further elucidated. However, few studies have explored the role of TGF-β2 and TGF-β3 in degenerative disc disease. Relatively speaking, more studies have focused on the anti-inflammatory effects of TGF-β or TGF-β1 isoforms in degenerative disc disease. Therefore, more evidence is still needed to support that TGF-β2 and TGF-β3 regulate the expression of TSLP in the intervertebral disc. The pro-inflammatory chemokine RANTES is significantly associated with IL-1β [65]. TNF-α and IL-1β regulate the secretion of the chemokine CCL3 in intervertebral disc cells through the MAPK, NF-κB, and C/EBPβ pathways [77]. TNF-α produced by macrophages is a potent inducer of MCP-1 expression [68]. In the presence of macrophages, MMP3 produced by chondrocytes contributes to the release of chemokines, leading to further macrophage migration [69]. In conclusion, the autoimmune response induced by herniated NP tissue is one of the most important mechanisms of LDH reabsorption. Macrophage infiltration in autoimmune responses is essential for LDH reabsorption to occur. Chemokines produced by lymphocytes and IVD induce macrophage migration to herniated IVD, and then inflammatory factors and chemokines secreted by macrophages act directly or indirectly to promote the recruitment of more macrophages. TSLP and MMP3 may be actively involved in this process by inducing chemokines. However, in many studies of reabsorption, despite the presence of chemokines in human IVD samples, there is still insufficient evidence (only a few animal models and in vitro studies) that chemokines can induce macrophage infiltration and reabsorption.

**Table 1:** Regulators involved in the recruitment of macrophages to the herniated disc

Regulator	Sample	Results	Reference
MCP-1, IL-8	Human IVD	Macrophages and capillaries have chemotaxis characteristics.	Burke et al. 2002 [60]
MCP-1, MIP-1α	Human IVD	They are overexpressed in macrophages, fibroblasts, and endothelial cells.	Haro et al. 1996 [37]
TSLP	Human and mouse IVD	Endogenous TGF-β reduces the expression of TSLP in the intervertebral disc by inhibiting NF-κB activation.	Zhu et al. 2013 [64]
RANTES, IL-1β	Human IVD	RANTES expression is upregulated by IL-1β.	Kepler et al. 2013 [65]
MCP-1	Rat IVD	MCP-1 promotes the absorption of rat nucleus pulposus tissue.	Haro et al. 2005 [66]
TSLP	Mouse IVD	TSLP induced by NF-kappaB activating ligand in the intervertebral disc may promote the recruitment of macrophages to the intervertebral disc by stimulating the production of MCP-1.	Ohba et al. 2008 [67]
MCP-1, MMP3	Mouse IVD	TNF-α induces the expression of MCP-1 and MMP-3.	Fujita et al. 2012 [68]
MMP3, MMP-7	Co-culture system of mouse chondrocytes and macrophages	MMP-3 produced by chondrocytes stimulates the production of macrophage chemoattractants.	Haro et al. 2000 [69]
TNF-α, IL-1β, CCL3	Rat IVD	TNF-α and IL-1β regulate the expression of CCL3 in nucleus pulposus cells.	Wang et al. 2013 [70]

Abbreviations: *MCP-1* monocyte chemoattractant protein 1, *IL* interleukin, *MIP-1α* macrophage inflammatory protein-1 α, *TSLP* thymic stromal lymphopoietin, *RANTES* chemokine ligand 5, *MMP* matrix metalloproteinase, *TNF-α* tumor necrosis factor-α, *CCL3* C-C Motif Chemokine Ligand 3

#### Inflammatory cascades associated with macrophages

##### Secrete inflammatory mediators

When herniated IVD triggers an autoimmune response, increased expression of chemokines, such as MCP-1, stimulates the recruitment of macrophages to IVD. The interaction of macrophages with IVD induces the production of inflammatory cytokines (Table 2) [85]. Results from several cohort studies suggested that serum inflammatory cytokine levels are unbalanced in LDH patients [78, 80, 86, 87]. Histological analysis of IVD in LDH patients revealed higher levels of expression of the inflammatory mediators TNF-α, IL-1β, IFN-γ, and IL-6 in extruding or sequestering IVD [58, 82, 83, 88, 89]. The in vitro IVD-macrophage co-culture model clearly revealed the interaction between macrophages and IVD. Co-culture conditions can induce high expression of IL-6 [84]. In addition, the expression of TNF-α was upregulated at the initial stage of co-culture, followed by the upregulated expression of IL-6, IL-8, and PGE2. Furthermore, TNF-α-induced production of IL-6 and PGE2 and IL-8 is independent of TNF-α stimulation, suggesting that there may be an inflammatory cascade that is independent of the TNF-α pathway [77]. In both studies, the cytokines were mainly produced by macrophages. In addition, related animal models have also explored signaling pathways that promote the upregulation of inflammatory mediators. A rat model of LDH demonstrated that increased expression of IL-1 and IL-6 was attributed to activation of the PI3K/AKT signaling pathway [90]. p38 MAPK can be activated by TNF-α and IL-1β secreted by macrophages or IVD [91, 92]. In addition, activation of the p38 pathway can also be involved in radicular pain by promoting the expression of inflammatory mediators [93].

**Table 2:** Inflammatory cytokines that regulate LDH

Inflammatory cytokines	Sample	Results	Reference
IL-6, IL-8, TNF-α, IL-4, IL-10	Patient serum	Sciatica is accompanied by an imbalance in the expression levels of inflammatory cytokines.	Wang et al. 2016 [78]
TNF-α, IL-4	Patient serum	The degree of pain is negatively correlated with the level of the anti-inflammatory cytokine IL-4.	Zu et al. 2016 [79]
IL-6, IL-8	Patient serum	IL-6 and IL-8 levels are elevated.	Pedersen et al. 2015 [80]
IL-6, COX-2, MMP-1, MMP-3	Human IVD	IL-1β upregulates the expression levels of IL-6, COX-2, MMP-1, and MMP-3.	Jimbo et al. 2005 [81]
TGF-β1, IGF-1, IL-6, IL-6R	Human IVD	The herniated IVD produces factors such as TGF-β1, IGF-1, IL-6, IL-6R, and fibronectin.	Specchia et al. 2002 [82]
IL-1α, IL-1β, IL-6, TNF-α	Human IVD	The herniated IVD produces inflammatory cytokines such as IL-1α, which in turn increase PGE2 production.	Takahashi et al. 1996 [83]
TNF-α, IL-6, IL-8, PGE2	Co-culture system of IVD and macrophages	TNF-α induces the production of IL-6 and PGE2, not IL-8.	Takada et al. 2012 [77]
IL-6	Co-culture system of IVD and macrophages	The infiltration of macrophages into the herniated disc may be a trigger for IL-6 production and related neurological symptoms.	Takada et al. 2004 [84]

Abbreviations: *COX-2* cyclooxygenase-2, *PGE2* prostaglandin E2, *TGF-β1* transforming growth factor beta-1, *IGF-1* insulin-like growth factor-1, *IL-6R* interleukin 6 receptor

Macrophages play a key role in LDH-mediated inflammatory responses. On the one hand, macrophages are involved in regulating inflammatory response, IVD degeneration, and sciatica; on the other hand, macrophages are also involved in spontaneous resorption. Some controversial conclusions can be attributed to the high plasticity of macrophages. After IVD injury, macrophage precursors are recruited to the herniated region by chemokines. These macrophages undergo phenotypic and functional differentiation under the influence of local tissue cytokines. The different phenotypes are divided into the classically activated M1 type and the alternatively activated M2 type. M1-type macrophages are characterized by their production of high levels of pro-inflammatory cytokines, such as TNF-α, IL-6, and IL-1β, which are closely associated with disc degeneration and sciatica, and are able to modulate the inflammatory response that mediates pain. In addition, some pro-inflammatory factors, such as TNF-α, can also stimulate the intervertebral disc to produce chemokines, induce the activation of matrix metalloenzymes, and indirectly promote the growth of new blood vessels, which is called functional inflammatory response. This is beneficial for the resorption of the herniated disc. This discrepancy in inflammation response is reflected in the inconsistent findings regarding the correlation between the presence of macrophages in herniated disc material and clinical symptoms [94]. The function of M2-type macrophages is to be anti-inflammatory and regulate wound healing. It plays a role in tissue repair, fibrosis, and tissue regeneration by modulating functional inflammatory effects. Studies have shown that anti-inflammatory cytokines secreted by these M2-type macrophages, such as IL-4 and IL-10, stimulate herniated disc resorption by promoting phagocytosis, as well as attenuating inflammatory responses [78, 95]. Currently, macrophage populations with mixed phenotypes have been identified in human herniated discs, which may lead to ideal NP tissue resorption and suboptimal inflammatory peripheral neuropathy manifesting as sciatica. Therefore, the promotion of intervertebral disc resorption by macrophage infiltration may be mainly attributed to the initiation of immune inflammatory response and phagocytosis. Besides, the promoting effects of M1 and M2 macrophages on reabsorption may alternate [96].

Some scholars have speculated that when there is modic change in the intervertebral disc, macrophages change from M2 type to M1 type, which in turn produces more pro-inflammatory factors, such as IL-6, IL-8, and TNF-α [96, 97]. Modic changes may represent a shift in the inflammatory response from “functional” to “painful,” thereby reducing recovery rates in LDH patients. Djuric et al. showed that there is an interaction between modic changes and the inflammatory response of the intervertebral disc regulated by macrophages [98]. However, the evidence to support this theory is currently very limited.

The different effects produced by macrophage infiltration depend on the type of macrophage in IVD. In most inflammatory processes, M1 and M2 macrophages often coexist. Their complex interactions result in mixed phenotypes that can activate or suppress regulatory effects in response to different stimuli [99]. Precise control of the phenotype of tissue macrophages is critical for tissue repair after injury [100]. Excessive M1-type macrophage polarization can lead to severe inflammation, severe tissue damage, and poor recovery. Excessive M2-type macrophage polarization may lead to insufficient inflammatory response and incomplete clearance of pathogens and cellular debris. Therefore, in the future, we need to explore the differentiation mechanism of macrophages recruited into the intervertebral disc and novel intervention methods, such as regulating the microenvironment of the herniated disc, and using epigenetic regulators to induce macrophage differentiation. This will help to better elucidate the biological mechanism of LDH reabsorption and relieve sciatica symptoms.

##### Induce the activation of matrix metalloproteinases (MMPs)

Matrix metalloproteinases contain 20 zinc-dependent enzymes that promote tissue uptake and remodeling of the extracellular matrix. Immunohistological analysis revealed that infiltrating macrophages and chondrocyte-like cells contained highly expressed matrix-degrading enzymes such as MMP-1, MMP-3, and MMP-7 in IVD (Table 3) [104]. The production of MMPs in IVD is mediated by cytokines secreted after macrophage infiltration. Inflammatory cytokines such as IL-1 and TNF-α are potent inducers of various MMPs including MMP3 and MMP-7. Increased expression of TNF-α in LDH-induced inflammatory responses leads to upregulation of plasmin, which subsequently activates MMPs. Anti-TNF-α antibodies can inhibit the activity of MMP3 [85, 101]. MMPs are thought to contribute to IVD degradation and resorption [103, 107, 108]. MMPs play two roles in promoting IVD resorption. On the one hand, upregulation of MMP expression upon induction of TNF-α accelerates the loss of proteoglycans and wet weight in herniated IVD, directly promoting the degradation of cartilage matrix, which is similar to their role in arthritic cartilage degeneration [66, 109, 110]. On the other hand, the secretion of MMPs is involved in the complex interaction of macrophages and chondrocytes in LDH reabsorption. Haro et al. provided important evidence that MMPs mediate LDH reabsorption. In a co-culture system of mouse macrophages and intervertebral discs, MMP-7 and TNF-α produced after macrophage activation promoted the release of soluble TNF-α (sTNF-α). sTNF-α is required for MMP-3 production by chondrocytes under co-culture conditions, and MMP3 expression is upregulated to release macrophage chemoattractants (e.g., MCP-1), which stimulate macrophage infiltration, proteoglycan loss, and intervertebral discs resorption. In addition, the induction of MMP-3 by TNF-α alone in the intervertebral disc is insufficient to generate chemoattractants and promote intervertebral disc resorption, suggesting that there may be other macrophage regulators that cooperate with TNF-α to mediate the release of chemokines regulated by MMP3 [69, 106]. In conclusion, macrophage infiltration is a key mechanism essential for disc resorption. The contact between macrophages and the intervertebral disc may initiate a cascade between macrophages/chondrocytes. MMP3 and MMP7 play a role in macrophage-chondrocyte communication, which indirectly affects the role of cartilage matrix degradation and intervertebral disc resorption. Furthermore, based on the critical role of MMP-7 in the process of LDH reabsorption and the nucleolytic effect of recombinant human MMP-7 (rhMMP-7) in human and canine intervertebral discs, Haro et al. developed rhMMP-7 intradiscal therapy [111, 112]. This therapy avoids the side effects associated with surgery, such as neurological deficits [111]. Currently, the therapy is in phase I/II clinical trials in the USA.

**Table 3:** Mediators involved in enzymatic degradation reactions during LDH reabsorption

Regulator	Sample	Results	Reference
MMP-1, MMP-3	Human IVD	Compared with MMP-1, MMP-3 is more conducive to LDH reabsorption.	Genevay et al. 2009 [101]
MMP-1, MMP-3	Human IVD	MMP-3 promotes degeneration of cartilage endplates.	Kanemoto et al. 1996 [102]
MMP-3, MMP-7, MMP-8	Human IVD	MMP-3, MMP-7, and MMP-8 play a role in nucleus pulposus reabsorption.	Haro et al. 1999 [103]
rhMMP3, rhMMP7	Human/rabbit/dog IVD	rhMMP-7 promotes LDH reabsorption.	Haro et al. 2005 [66]
MMP-1, MMP-3	Human IVD	The expression of MMP-1 and MMP-3 is increased in inflammatory cells of IVD granulation tissue.	Matsui et al. 1998 [104]
MMP-1, MMP-3	Co-culture of human IVD and monocytes	Cytokine stimulation helps promote the production of matrix metalloproteinases in intervertebral disc cells.	Doita et al. 2001 [105]
MMP-3, MMP-7, TNF-α	Co-culture system of mouse IVD and macrophages	MMP-7 produced by macrophages is required for proteoglycan degradation and macrophage infiltration.	Haro et al. 2000 [106]

*Abbreviations*: *rhMMP* recombinant human matrix metalloproteinase

##### Promote the formation of new blood vessels

Vascular ingrowth has been recognized as an essential feature of spontaneous LDH absorption [113, 114]. Histological examination revealed neovascularization at the site of a herniated disc [114, 115]. Furthermore, enhanced MRI showed that herniated IVD fully exposed to the epidural space was resorbed more frequently, which was positively correlated with vascularization [116]. Mediators that induce LDH neovascularization mainly include TNF-α, VEGF, basic fibroblast growth factor (bFGF), and platelet-derived growth factor (PDGF) [30, 117]. The activity of different types of macrophages and the secreted pro-angiogenic mediators are the main regulators of neovascularization in the inflammatory response. As described above, macrophages are classified into M1 type and M2 type after activation. M2 macrophages can be further subdivided according to their function. Macrophages activated by IL-4 that mediate wound healing belong to the M2a subtype; regulatory macrophages activated by immune complexes, glucocorticoids, and IL-10 are of the M2c subtype. Spiller et al. [118] proposed a potential mode of interaction between M1 and M2 macrophages in regulating angiogenesis. Early M1 macrophages promote vascular sprouting by secreting VEGF, bFGF, IL8, RANTES, and TNF-α. The pro-angiogenic effect of M2c subtype macrophages is manifested in promoting vascular remodeling by secreting high levels of MMP-9. M2a subtype macrophages promote vascular fusion through an unknown secreted factor. M2a macrophages can also regulate the function of M1 macrophages by producing tissue inhibitor of metalloproteinase 3 (TIMP3) and recruit pericytes to promote the maturation of new blood vessels by secreting platelet-derived growth factor-BB (PDGF-BB).

The relationship between macrophages and angiogenesis in LDH has attracted the attention of some researchers. In a co-culture model of macrophages and IVD, macrophages act as pro-vascularization mediators in the IVD microenvironment. Haro et al. [30] found that VEGF expression levels were increased in IVD-macrophages and the induction of VEGF is dependent on TNF-α. Next, Ohba et al. also demonstrated that TNF-α regulates VEGF expression; TNF-α in herniated intervertebral disc mainly induces the expression of VEGF in intervertebral disc cells through the NF-kappaB pathway, thereby promoting angiogenesis [119]. Taken together, these studies highlighted the involvement of macrophage infiltration into IVD in regulating angiogenesis. During this process, macrophages may undergo a transformation from M1 to M2 type. Cytokines secreted early by M1 macrophages may initiate angiogenesis, while M2 macrophages promote endothelial cell proliferation and stabilize blood vessel growth by secreting MMP9 and PDGF-BB.

In addition, other factors are also involved in the formation of blood vessels in the intervertebral disc. Johnson et al. found that human IVD proteoglycan inhibited endothelial cell adhesion and migration in vitro [120], and mechanical stimulation could alter proteoglycan expression and affect its ability to promote endothelial cell migration [121]. This indicates that human IVD proteoglycan can also affect the ingrowth of blood vessels and indirectly regulate the resorption of herniated intervertebral discs. Moon et al. suggested that annuli cells may stimulate the expression of MMPs by secreting IL-8 and VEGF, thereby promoting angiogenesis and invasion [122]. Furthermore, in herniated IVD, neovascularization also provides a hematogenous pathway for macrophage invasion. Thus, there is a complex crosstalk between macrophage recruitment, inflammatory responses, and neovascularization.

It is worth noting that, in order to develop therapies to promote LDH reabsorption, some studies established a rabbit model of free LDH to explore the effects of cytokines and drug interventions on angiogenesis during LDH reabsorption. Epidural injection of bFGF may promote resorption of herniated discs by stimulating angiogenesis [40]. Epidural injection of midkine (MK) promotes neovascularization, degrades the intervertebral matrix, and facilitates infiltration of inflammatory cells. MK is considered to be a chemokine for neutrophils [123]. The production of MK may promote lymphocyte infiltration into the herniated disc, promote neovascularization, and promote the inflammatory cascade, which in turn promotes LDH reabsorption. Compared with epidural steroids, lipopolysaccharide promotes inflammatory cell accumulation and neovascularization, which in turn promotes LDH reabsorption [124]. Most of the current rabbit LDH models are autograft models. Compared with the rodent tail models, the rabbit LDH model has higher homology to human discs, has larger animal and disc sizes, and is more economical than larger animals [125]. Compared with in vitro cell models, the advantage of autologous transplantation models is that the microenvironment in which cells exist in the extracellular matrix is preserved. Notochordal cells in human discs gradually disappear after human adulthood, while notochordal cells in rabbit intervertebral discs are preserved after adulthood [126]. Persistence of notochord cells is an important factor, which generates intervertebral discs with different biomechanical and biological properties [126]. Although animal discs that retain notochordal cells help us understand the cellular biology of the human nucleus pulposus during disc maturation, the experimental results obtained with the model may not be applicable to adults and should be treated with caution. Studies have reported that some animals (chondrodystrophic dogs, old sheep, and cattle) lose notochord cells at some point after skeletal maturity, which may serve as a promising animal model for human intervertebral disc herniation [125–129]. Recently, Rawson et al. proposed a new therapy [130], which involves injection of platelet-rich plasma (PRP) into the lumbar spine and epidural injection of platelet lysate (PL), which can initiate or accelerate the resorption of the herniated lumbar disc. This effect relies on a complex interplay of cytokines and growth factors that promote neovascularization and macrophage-induced disc phagocytosis. This study described 2 symptomatic LDH patients who experienced significant improvement in symptoms after epidural injection of concentrated growth factors from platelet lysates. However, the study does not clearly identify which growth factors mediate reabsorption. To further confirm the efficacy and safety of PRP/PL therapy, investigators should provide more case reports, explore growth factors and optimal concentrations that promote intervertebral disc absorption, and conduct randomized double-blind trials and safety trials.

#### Other mechanisms involving resorption of herniated discs

The reabsorption of LDH is a complex physicochemical process. The known classical biological mechanisms involve the regulation of various cytokines after macrophage infiltration into the intervertebral disc tissue, including phagocytosis, inflammatory response, matrix degradation, and neovascularization. Besides, the biological mechanism of LDH reabsorption may also involve apoptosis and autophagy of nucleus pulposus cells.

In the rat LDH model, our group observed that when the herniated IVD was ruptured or dissociated, the rate of apoptosis was higher than that of the unruptured and normal IVD [131]. Among the cytokines released by macrophage infiltration, some cytokines, such as Fas, IL-2, and HIF-α, have been shown to induce apoptosis of intervertebral disc nucleus pulposus cells, thereby promoting LDH reabsorption [132–134]. p38MAPK is an important signaling pathway mediating nucleus pulposus cell apoptosis. Wang et al. [135] found that IL-2 induces apoptosis in NP cells and activates the p38 MAPK signaling pathway. Activated p38MAPK signaling pathway induces apoptosis in NP cells and accelerates the reabsorption of ruptured LDH [131].

In addition, autophagy is closely related to apoptosis, immune response, and inflammatory response. Autophagy levels within the physiological range can protect cells and maintain body stability, while excessive autophagy can cause autophagic death of cells [136]. Some scholars believe that autophagy creates favorable conditions for delaying intervertebral disc degeneration and reabsorption [137–139]. Ma [138] et al. found that ROS-mediated autophagy promotes degeneration in rat nucleus pulposus cells after stress stimulation. Stress stimulation increases Beclin1 levels and promotes the processing of LC3B-I into LC3B-II, a key step in the formation of “autophagosomes.” Studies have demonstrated that autophagy protects endplate cells from calcification, and the stimulation of endplates is derived from intermittent cyclic mechanical tension [140]. In a state of “serum deprivation” of starvation, hypoxia favors nucleus pulposus cell survival by downregulating excessive levels of autophagy [141]. Although there are no related studies on autophagy and intervertebral disc reabsorption, we can learn from some changes of autophagy under hypoxica and stress, because disc degeneration or reabsorption in this condition involves immune response and inflammatory response. Exploring how to effectively regulate autophagy to promote intervertebral disc absorption will provide new ideas for clinical treatment of LDH. Thus, we need more direct evidence to elucidate the role and mechanism of autophagy on intervertebral disc reabsorption.

#### Discussion

**Fig. 2:**
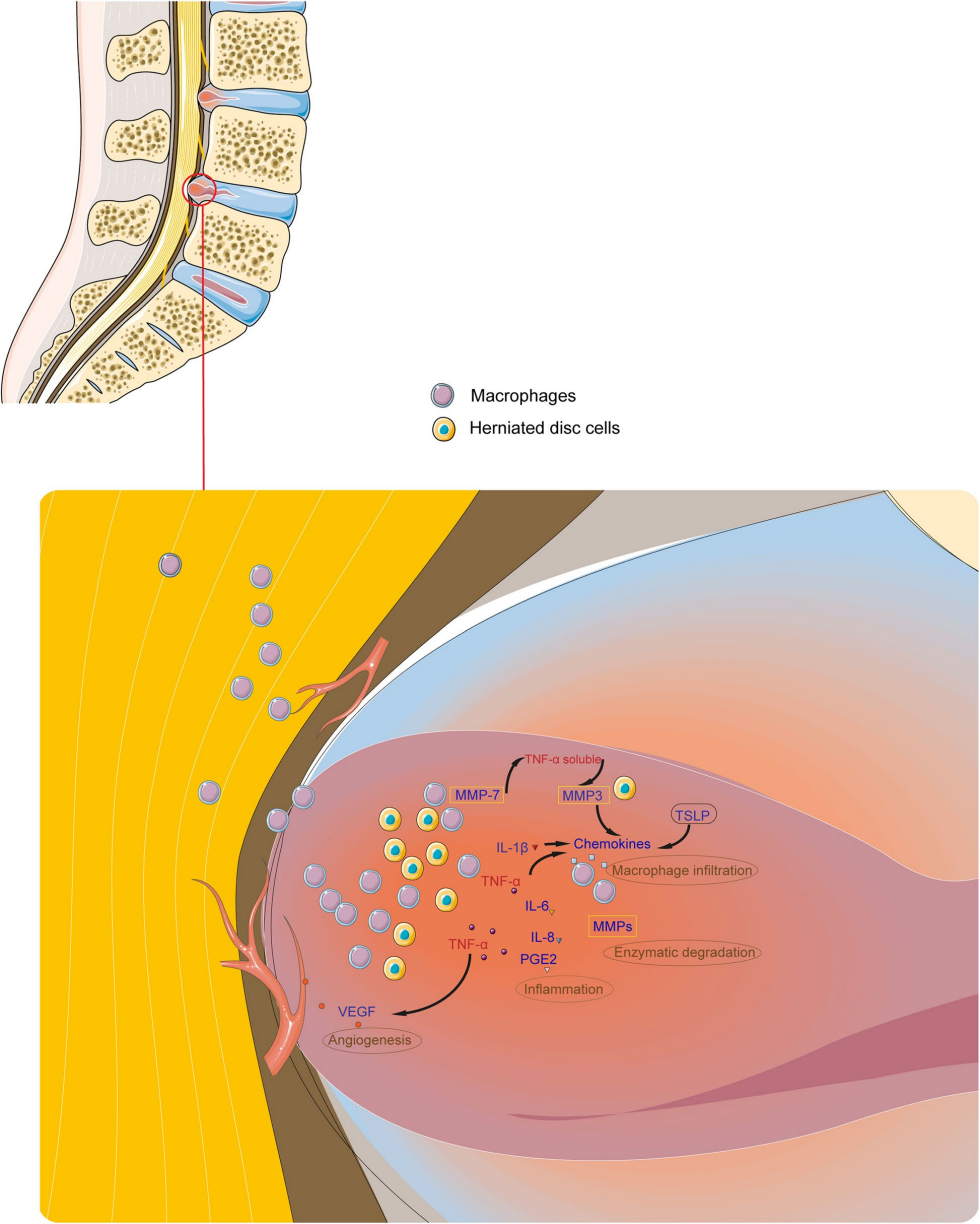
The main mechanism of reabsorption of herniated disc. A herniated intervertebral disc causes macrophages to infiltrate the intervertebral disc tissue, and the macrophages interact with the intervertebral disc cells to release TNF-α, VEGF, IL-8, IL-6, IL-1β, MCP-1, and MMPs. These cytokines interact to promote resorption of the herniated disc. The illustration elements are from Servier Medical Art (https://smart.servier.com/)

LDH reabsorption is the result of the sequential occurrence and interaction of multiple physiological processes (Fig 2). When protruding IVD tissue squeezes out of the epidural space, it disrupts immune privilege, triggering an autoimmune response, then lymphocytes activate macrophages. Related factors secreted by IVD cells and macrophages further drive the recruitment of macrophages to the intervertebral disc in paracrine and autocrine forms. Macrophages undergo differentiation from M1 to M2 types. M1-type macrophages secrete pro-inflammatory factors to initiate angiogenesis, promote the expression of matrix metalloenzymes and apoptosis of herniated IVD nucleus pulposus cells. M2-type macrophages secrete anti-inflammatory factors to relieve pain response, promote new blood vessel formation, and are responsible for tissue remodeling and repair, and absorb the protruding debris to reduce the total volume of the intervertebral disc, and reduce the mechanical compression of the nerve. Throughout the process, infiltration and activation of macrophages mediate inflammatory responses, matrix metalloenzyme activation, and neovascularization. Clinical studies mainly rely on imaging and histology to observe macrophage infiltration, angiogenesis, and inflammatory factors. We still lack suitable models to probe the immune responses involved in reabsorption. Current basic research models include in vitro co-culture models and animal models. In vitro co-culture models often only have a single stimulus and cannot truly simulate the complex microenvironment of human LDH. Most animal models are autologous transplantation models. The IVD is transplanted into the subcutaneous or dorsal epidural space of the animal after excision. The advantage of this model is that it is simple to operate, but the disadvantage is that the rat, a non-upright animal, cannot mimic the biomechanical environment of human LDH. This model also failed to reproduce the clinical symptoms of radicular pain. In addition, there is controversy about how LDH induces an inflammatory response. There are three possibilities, one is through exposure to structural elements and compounds in the intervertebral disc cell membrane and matrix, the second is through direct contact of NPs with the immune system, and the third is secondary to an autoimmune reaction. Therefore, it is still necessary to explore and develop animal models that are highly similar to human LDH in terms of biomechanics, microenvironment, mechanics, and cell phenotype/distribution, which will help clinicians to better understand the biological mechanisms that promote LDH reabsorption.

## Current treatments for LDH

Although ruptured LDH reabsorption is a natural history, we can promote the reabsorption process through clinical interventions. A large number of clinical studies have shown that it is beneficial for LDH patients to choose reasonable non-surgical treatment to improve clinical symptoms and accelerate the reabsorption of protrusions.

Pain relief by controlling inflammation is a key strategy in the treatment of LDH, but administration of anti-inflammatory drugs may be detrimental to LDH reabsorption. However, there are some case reports that the use of oral non-steroidal anti-inflammatory drugs (NSAIDs) or intraspinal steroids can induce protrusion reabsorption, which may be related to the reduction of local inflammatory edema. Based on the inflammatory mechanism of LDH reabsorption, the early inflammatory response is involved in recruiting macrophages and inducing factors that promote reabsorption. Currently, commonly used NSAIDs or intraspinal steroids control pain by suppressing local inflammation, but long-term use of these drugs may hinder reabsorption. A prospective study by our group has shown that anti-inflammatory drugs hinder LDH reabsorption [142]. In addition, most traditional Chinese medicines have little toxicity and side effects, and are mostly applied in the form of compound prescriptions, which can alleviate intractable diseases through reasonable compatibility [143–145]. Studies have shown that traditional Chinese medicine therapy shows the possibility of promoting LDH reabsorption and improving clinical symptoms [131, 146–148], but more clinical trials are still needed. In addition, the edema caused by the local inflammatory response at the disc herniation can be alleviated with dehydrating agents [149]. There are two potential mechanisms of dehydrating agents, one is to change the compression relationship between the nerve root and the herniated intervertebral disc by reducing the edema of the nerve root [149]. The other is that dehydrating agent may act on a part of the herniated nucleus pulposus with high water content, that is, promote the dehydration and atrophy of the herniated nucleus pulposus. But the above mechanism is only a hypothesis, lacking solid experimental evidence. In the future, our team will continue to study the biological mechanism of LDH reabsorption and conduct large-scale and standardized clinical trials to evaluate the effectiveness of conservative therapy in promoting LDH reabsorption.

Traditional strategies for LDH such as drug therapy, injection of epidural steroids, and surgery all carry potential risks of iatrogenic sequelae. Based on the biological mechanisms of protrusion reabsorption, such as macrophage infiltration, protrusion vascular ingrowth, and matrix metalloproteinase activation, researchers have proposed new potential therapies to promote LDH reabsorption. Epigenetic modulators and ozone intradiscal injection therapy can interfere with the differentiation of macrophages, thereby reducing inflammation and pain, and promoting resorption of the nucleus pulposus. Epidural injection of pro-angiogenic factors can promote new blood vessel formation and induce macrophage phagocytosis. Chemical nucleolytic therapy with rhMMP-7 reduces the water content of the LDH matrix by degrading aggrecan and/or collagen, thereby inhibiting nerve compression by the protrusions. Future studies should focus on balancing macrophage differentiation, rationally controlling inflammatory responses and promoting angiogenesis. There is an urgent need to develop new clinical treatments, increase safety studies, and explore the growth factors and their optimal concentrations needed to promote intervertebral disc absorption. Besides, randomized, double-blind, controlled trials are necessary for demonstrating the efficacy of new therapies in promoting LDH reabsorption.

## Conclusion

LDH is one of the most common diseases in the spine. With the support of radiology technology, clinicians can scientifically and reasonably diagnose disc herniation diseases, and then provide patients with feasible and effective treatment strategies. Clinical studies have shown that the possibility of spontaneous absorption of LDH without surgical treatment is higher. The type and composition of the herniated disc could predict the possibility of natural resorption of the herniated disc. Therefore, these can be considered as factors for clinical diagnosis and treatment. Extrusion and sequestration LDH can squeeze out of the epidural space, creating favorable conditions for macrophage infiltration and neovascularization. These two types of LDH are important basis for clinicians to judge whether LDH reabsorption occurs. In addition, if the disc herniated tissue contains a higher proportion of cartilage or shows Modic changes, it is not conducive to macrophage infiltration and ingrowth of blood vessels, thereby preventing the occurrence of LDH reabsorption. To be sure, when there is no indication for surgery and the tissue does not show Modic changes, regardless of the size of the protrusion, a variety of conservative treatments should be given priority instead of surgery.

We summarized the literature on the biological mechanisms of LDH reabsorption and highlighted the critical role of autoimmune responses in spontaneous disc resorption, including inflammatory responses mediated by macrophage infiltration interacting with the disc, enzymatic degradation responses, and angiogenesis. Based on the biological mechanism of LDH reabsorption, we can propose new clinical treatments for LDH. Future studies can focus on secreted factors from macrophages and IVD cells that may be involved in macrophage recruitment/differentiation, activation of matrix metalloenzymes, and neovascularization, which are potential therapeutic targets for promoting LDH reabsorption.

## Abbreviations

LDH: Lumbar disc herniation
MCP-1: Monocyte chemoattractant protein 1
IL: Interleukin
TSLP: Thymic stromal lymphopoietin
MMP: Matrix metalloproteinase
TNF-α: Tumor necrosis factor alpha
PGE2: Prostaglandin E2
COX-2: Cyclooxygenase 2
rhMMP: Recombinant Human Matrix Metalloproteinase
